# Analysis of rural health centres preparedness for the management of diabetic patients in Malawi

**DOI:** 10.1186/s13104-018-3369-7

**Published:** 2018-05-02

**Authors:** Ibrahim Chikowe, Victor Mwapasa, Andre Pascal Kengne

**Affiliations:** 1 Biomedical Sciences and Pharmacy Departments, College of Medicine, University of Malawi, Blantyre, Malawi; 2 Department of Public Health, College of Medicine, University of Malawi, Blantyre, Malawi; 3 Non Communicable Diseases Research Unit, South African Medical Research Council & University of Cape Town, Capetown, South Africa

**Keywords:** Diabetes, Management, Health center, Medicines, Non-communicable diseases, Malawi

## Abstract

**Objective:**

There is limited data on the quality of primary care management for diabetes mellitus across Africa. The study was aimed at assessing the availability of basic supplies for the rapid diagnosis, treatment and management of diabetes in Malawian rural health facilities. This cross-sectional study was conducted in 55 public and private health centers from 19 districts using a structured questionnaire and checklist to interview the pharmacy personnel or officer in-charge of the health centers. We focused on availability of information, diagnosis and treatment materials for diabetes.

**Results:**

Of the 55 health facilities surveyed, 21, 23 and 11 were located in the central, southern and northern regions of Malawi, respectively. Overall, 38% (21/55) of the health centres had glucometers, while 24% (13/55) had urine glucose dipsticks. Only 4% (2/55) of the health centres had recommended first-line medicines for treatment of type 1 and type 2 diabetes. No health centre had diabetes patient records and information, education and communication materials. Most rural health centers in Malawi lack basic health commodities for the screening, diagnosis and treatment of diabetes and this impedes on their effective management of growing diabetes burden. Therefore, health care systems need to adequately equip primary care facilities.

**Electronic supplementary material:**

The online version of this article (10.1186/s13104-018-3369-7) contains supplementary material, which is available to authorized users.

## Introduction

Diabetes mellitus is one of the chronic non-communicable diseases (NCDs) with rapidly increasing morbidity and mortality in sub-Saharan Africa and worldwide [1]. Established evidence-based guidelines for management of diabetes mellitus and quality of care are still underdeveloped in many developing countries [2, 3]. The magnitude and infrastructure for NCDs care, preparedness and quality of services in sub-Saharan Africa has been questioned, but not widely documented [4, 5]. Studies are therefore needed to provide data for evidence-based policies and adequate response at primary care level for patients with NCDs [6, 7]. In Malawi, health facilities have had challenges in coping with common diseases, including NCDs, making effective case management difficult particularly in underserved rural areas [8]. This study therefore assessed the availability of basic supplies for the rapid diagnosis, treatment and management of diabetes in rural health facilities in Malawi.

## Main text

### Methods

#### Study setting

**Fig. 1:**
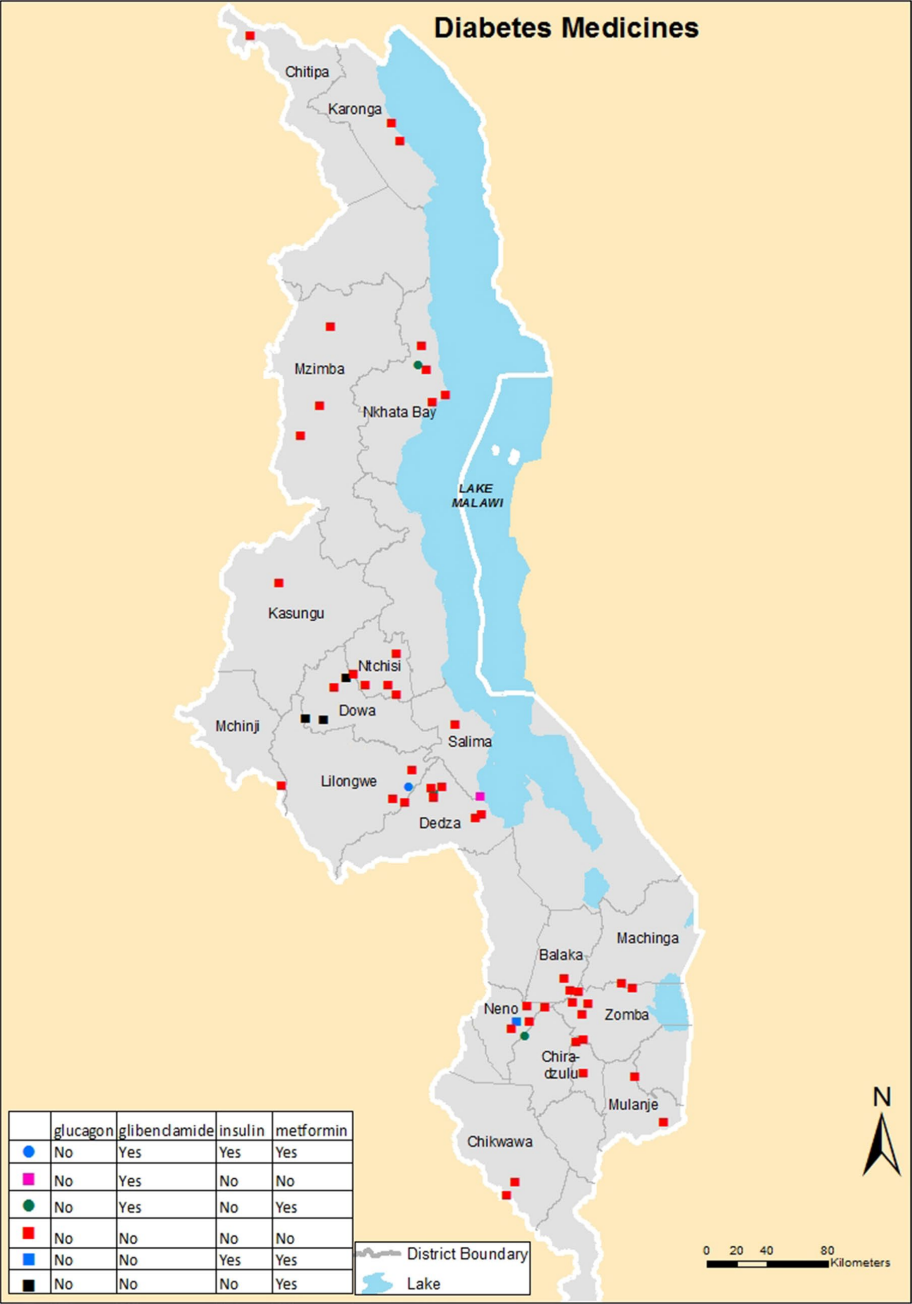
Map showing spatial distribution of health centres with different combinations of drug regimens available per health centre

This was a cross-sectional study conducted between November, 28 and December, 31 2016 on 55 health centers from 19 of 28 districts in Malawi. Majority of health centres were owned by government (n = 42) and the rest by Christian Health Association of Malawi (CHAM, n = 9), Non-Governmental Organisations (NGOs, n = 3) and a private entity (n = 1) getting essential medical supplies support from government for the surrounding communities they serve for free. Out of the 55 health centres, 21 (38.2%) were from southern region, 23 (41.8%) from central region and 11 (20.0%) from northern region. Figure 1 below shows distribution of the selected health facilities countrywide (Additional file 1: Figure S1).

#### Data collection

Data were collected by investigators administering questionnaires to health care providers (HCPs) including pharmacy assistants/technicians, clinicians, nurses, health surveillance assistants (HSAs) and others knowledgeable about the pharmacy, diagnosis room and testing room operations. HSAs are community health workers recruited by government to implement preventive and health promotion activities at health facility and community levels [9]. Locations of the health facilities were recorded using global positioning system (GPS) equipment (Additional file 1: Figure S1). Data collectors also accessed stock cards, delivery notes and monthly reports. In addition, physical inventories were performed on medical supplies.

Data collection focussed on availability of information, education and communication (IEC) materials, medicines (Additional file 2: Figure S2), reagents and/or tools for screening, diagnosing and treating diabetes viz. glucometer, and urine dipstick (Table 1). The informants were asked whether a medical supply was available, enough (for population, facility departments and all designated users), functional or did not know.

The data collection tool was based on questionnaire items developed and adapted by Pakhare et al. [10] from Indian Public Health Standards [11], World Health Organization (WHO) package of essential NCD interventions guidelines [12] and the WHO Service Availability and Readiness Assessment manual (SARA) [13] (Additional file 3: Questionnaire). Data were analysed using Microsoft Excel and ArcGIS ArcMap 10.3.1 and include essentially descriptive statistics.

### Results

#### Characteristics of health facilities

Majority of the selected health centres (94.5%; 52/55) had shortage of qualified pharmacy assistants. Thus, in most facilities, HSAs, nurses, clinical officers/medical assistants were managing health facility drug stores/pharmacies. In exceptionally separate cases, a driver and a grounds labourer managed a drug store. Most of the HSAs had received some training for 3 days as drug store attendants while in others not. In some cases, officers in-charge of health facilities doubled as pharmacy technicians and this affected drug store management, including updating of pharmacy records, since most were too busy with their primary responsibilities. In places where HSAs managed drug stores, routine pharmacy activities were sometimes paralysed whenever they went for community activities.

#### Availability of equipment and supplies, information resources and medicines

Table 1 below shows the availability of health commodities at the health facilities.

**Table 1:** Number of health centres with health commodities for basic management of diabetes

Category of resources	Specific type of health commodity	Health centre (N = 55)	
		# of health centers with the commodity	Percent of health centers with the commodity (%)
Diagnostics	Urine dipsticks (urinary ketone strip/urinary protein strip)	13	23.6
	Glucometer	21	38.2
Medicines	Glibenclamide	5	9.1
	Metformin	8	14.5
	Insulin	1	1.8
IEC materials	Posters	0	0.0
	Patient records	0	0.0

#### Availability of basic diabetes diagnostic equipment and supplies

Just over one-third (38.2%) of the health centres had glucometers (Table 1). The proportions of health facilities with glucometers were 48% (10/21), 22% (5/23) and 55% (6/11) in the southern, central and northern region, respectively. Excluding non-functional glucometers, the number of health centres with glucometer in the southern, central and northern Malawi was 38% (8/21), 22% (5/23) and 18% (2/11), respectively.

Glucose urine dipsticks were available in less than one-quarter (23.6%) of the health centres countrywide (Table 1). The proportions of health facilities with dipsticks were 38% (8/21) in the south with 14% (3/21) only having functional ones. In the central, 22% (5/23) of health centres had dipsticks out of which 17% (4/23) were functional.

#### Availability of information, education and communication materials

Diagnostic and guidelines are supposed to be available in the consultation room and IEC materials to be in the waiting area. These materials were not where they were expected. In all health facilities, there were no visible information, education and communication (IEC) materials on NCDs prevention and management.

#### Availability of key medicines for diabetes management

**Fig. 2:**
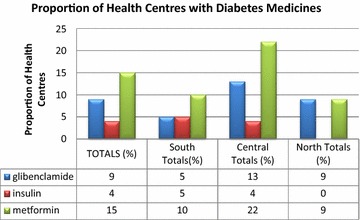
Number of health centres with diabetes medicines in the three regions

Figure 2 shows poor availability of diabetes medicines in the health facilities ranging from 0 to 22%, across the three regions. Metformin was available in approximately one-fifth (22%) of the facilities in the central region, but the proportions were much lower in the other regions. Glibenclamide was available at most 15% of the health facilities in the central region but, again, the proportions were lower in the northern and southern region.

Figure 1 below shows spatial distribution of the health centres with and without medicines for diabetes medicines. Each colour and shape shows the medicine or combination of medicines available and those not available.

### Discussion

#### Testing and diagnostic tools using point of care (POC) monitoring devices

Rural facilities lack sophisticated laboratories and need point of care (POC) devices for diagnosis of diabetes. Here, majority of health facilities lacked POC devices and this is consistent with findings from other sub-Saharan African countries; urine glucose strips were available in 18% and blood glucose meters in 21% of health-care facilities in Mozambique; 13% of health facilities had blood glucose meters and 54% had urine glucose strips in Mali; 49% and 61% had blood glucose and urine glucose strips, respectively in Zambia [1, 14]; 38% of health centres and 17% of dispensaries had diagnostic equipment in Tanzania [5]. Therefore, shifting to POC devices remains to solve the poor availability of diagnostics in clinics [1]. Lack of diagnostics may delay diagnosis, screening, health education and treatment initiation, treatment monitoring, regimen changes, prevalence data generation, rapid progression of disease to serious state and could be fatal for type 1 diabetes patients [15–18]. Furthermore, high prevalence of HIV/AIDs and malaria in sub-Saharan African countries can cause misdiagnosis of diabetes cases since some symptoms (rapid weight loss, fatigue, abdominal pain, and confusion) are similar [1].

#### Availability of medicines for diabetes

Availability of medicines for diabetic outpatients was generally poor and only two health centres had medications for both type 1 and 2 diabetes mellitus (insulin plus any other). This confirms Assayed et al. results that rural Malawians have limited access to diabetes treatment and diagnosis and they travel long distances of mostly at least 10 km to access the services [8]. The shortage of first-line regimens is also consistent with previous findings from WHO service availability and readiness assessment surveys in nine sub-Saharan countries Benin, Burkina Faso, Democratic Republic of Congo, Kenya, Mauritania, Sierra Leone, Tanzania, Uganda, and Zambia on availability of insulin, metformin, and glibenclamide. Insulin was available in 13% of the surveyed facilities (lower levels were reported in 12% and 7% median of primary care in rural and urban areas, respectively). The median availability of metformin glibenclamide was 22% which decreased in primary levels. In the absence of diabetic management facilities in rural facilities, patients’ only alternative are secondary and tertiary health facilities which are usually far [13]. With high poverty levels, they may forgo treatment monitoring that may lead to rapid onset of diabetic complications, reliance on traditional systems (spiritual healing, traditional medicines) and death [19, 20].

#### Availability of information, education and communication materials for diabetes

Patient records and IEC materials for diabetes were also not available in all health centres. The lack of IEC materials in health facilities found in this study is also consistent with previous findings from Tanzania in which the IEC were available in 25% of dispensaries and none of the health centres [5]. The WHO report also showed that IEC materials especially guidelines were available in a third of the facilities offering diabetes services in the nine surveyed countries in the sub-Saharan Africa [1, 13]. The lack of IEC materials threatens diabetes care, medicine safety and treatment [21] since services in majority of health centres in Malawi are provided by medical assistants, nurses and health surveillance assistants [22]. This also threatens the management of diabetes by patients and guardians since they are deprived of basic diabetes education (prevention, complications detection and healthy lifestyle) [1, 21]. The lack patient records deprive the health systems of sources of information for documentation of diabetes control, medications and complications [1].

The data suggests that the shortages could be due to lack of trained pharmacy personnel or limited orientation of clinicians to the management of diabetes. However, this study did not examine the whole supply chain for diabetes to pin-point the source of the problem. The results support what most studies have found that primary care health systems for NCDs remain weak in sub-Saharan Africa countries [5, 8, 23, 24].

Availability of diabetes medicines currently stands at 27% (15/55) of the rural health centres with at least one treatment regimen, while testing materials availability stands at 24% (13/55) of health centres with urine dipsticks and 38% (21/55) with glucometers. The current situation is not conducive for a chronic disease that needs proactive, patient-centred, community based and sustainable long-term undisrupted care [25, 26].

### Conclusion

This study found a small proportion of primary health facilities with adequate resources for screening and treating diabetes. It also highlighted the lack of IEC materials for clinicians and patients knowledge and promotion of diabetes management (prevention, screening and therapy) in all facilities. Thus, despite the increasing prevalence of diabetes in Malawi, health facilities were ill-equipped to combat this disease. The findings suggest the need to define a minimum package of medical and drug supply for diabetes management in primary care facilities. The ministry of health should focus on providing clinical guidelines for diabetes management, basic diabetes diagnostic equipment, first-line drug therapy for diabetes mellitus and establishing strong management systems for diabetes like regular training, supervision and reporting. These may support provision of personal health care needs to populace and development of sustainable partnership with patients.

## Limitations of the study

The sample size of the study may not have been since the facilities were not randomly selected. The study was also limited in terms of data collection process because of limited knowledge of some respondents about some study parameters assessed. The depth of the study was also limited as it did not assess the functionality of the referral system for diabetic patients as well as its focus on glucose, yet diabetes diagnosis and management go beyond glucose monitoring.

## Additional files

**Additional file 1: Figure S1.** Map showing spatial distribution of health centres with different combinations of drug regimens available per health centre

**Additional file 2: Figure S2.** Number of health centres with diabetes medicines in the three regions

**Additional file 3: Figure S3.** Questionnaire for the study.

## Abbreviations

AIDS: Acquired Immuno Deficiency Syndrome
CHAM: Christian Health Association of Malawi
GPS: Global Positioning System
HCP: Health Care Provider
HIV: Human Immunodeficiency Virus
HSA: Health Surveillance Assistant
IEC: Information, Education and Communication
NCD: Non-Communicable Disease
NGO: Non-Governmental Organisation
POC: Point of Care
WHO: World Health Organisation
