# Supplementary figures and images for: Analysis of rural health centres preparedness for the management of diabetic patients in Malawi
Source: BMC Res Notes. 2018 May 2;11:267. doi: 10.1186/s13104-018-3369-7 (PMC5932777; doi:10.1186/s13104-018-3369-7)

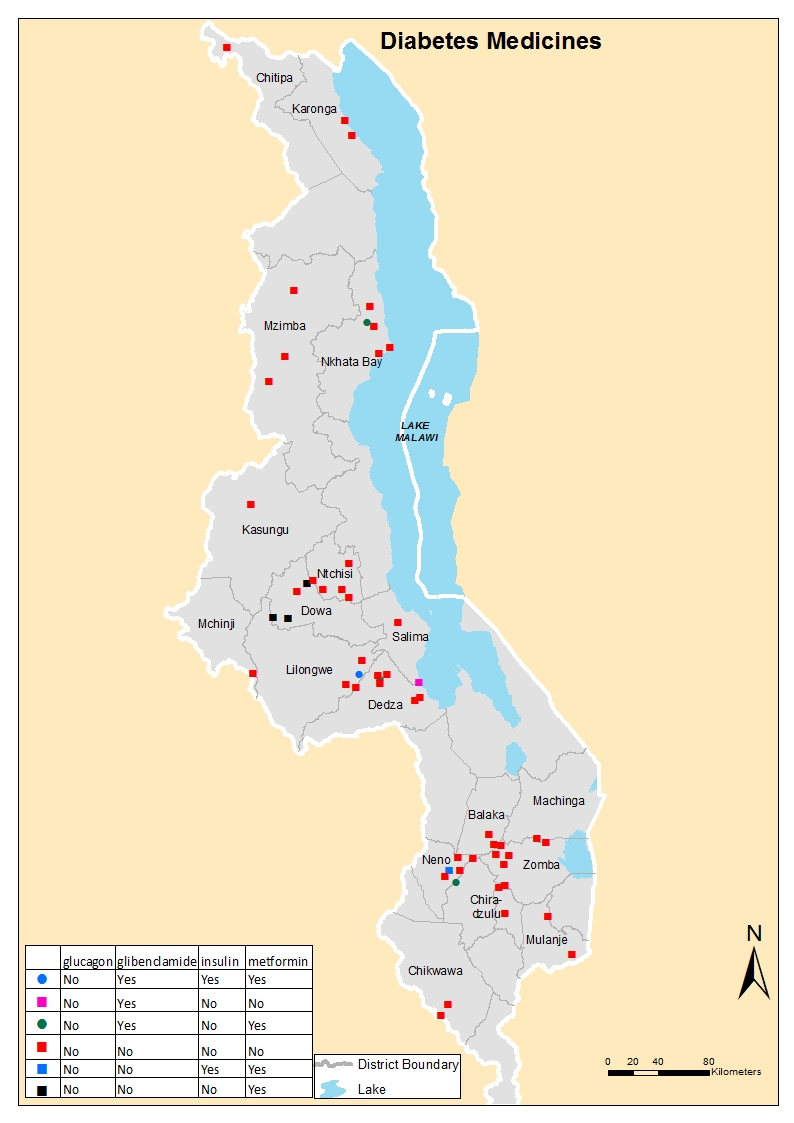

Supplement: Supplementary file 1 — Additional file 1: Figure S1. Map showing spatial distribution of health centres with different combinations of drug regimens available per health centre [file 13104_2018_3369_MOESM1_ESM.docx]
