# Supplementary material for: Analysis of rural health centres preparedness for the management of diabetic patients in Malawi
Source: BMC Res Notes. 2018 May 2;11:267. doi: 10.1186/s13104-018-3369-7 (PMC5932777; doi:10.1186/s13104-018-3369-7)
Supplement: Supplementary file 3 — Additional file 3: Figure S3. Questionnaire for the study. [file 13104_2018_3369_MOESM3_ESM.docx]

**Questionnaire**

**SECTION A**

1. **Institution:**
2. **Respondent Position:**
3. **Authority:** Mission NGO Government Government + NGO Other (specify)
4. **Setting:** Rural Urban
5. **Ceiling:** Y N
6. **Air conditioner:** Y N Functional: Y N
7. **Temperature:**

**SECTION B**

1. **Do you have the following?**
   1. **Stethoscope:** Y N DK Enough? Y N DK Functional? Y N DK
      1. Comment:
   2. **Glucometer:** Y N DK Enough? Y N DK Functional? Y N DK
      1. Comment:
   3. **BP monitor:** Y N DK Enough? Y N DK Digital? Y N DK Functional? Y N DK
      1. **Comment:**
   4. **Urine dipstick:** Y N DK Enough? Y N DK Functional? Y N DK
      1. **Comment:**
2. **Do you have the guidelines for the following diseases?**
   1. **Diseases** **(circle available):** Diabetes Cardiovascular diseases/Hypertension/Heart failure Respiratory diseases/COPD/Asthma Epilepsy Cancer
3. **Do you have the following medicines (circle the available one):**
   1. **Antiepileptics:** magnesium sulphate, midazolam, phenobarbital, phenytoin, diazepam, valproic acid/sodium valproate, ethosuximide, carbamazepine, paraldehyde, thiamine
      - 1. Other:
   2. **Cardiovascular medicines:** nifedipine, bisoprolol, morphine, gylyceryl trinitrate, isosorbide dinitrate, aspirin, verapamil, digoxin, epinephrine, adrenaline, lidocaine, amiadorone, amlodipine, enalapril, hydralazine+nitrate, hydrochlorothiazide, methyldopa, sodium nitroprusside, frusemide, bendrofluazide, spironolactone, dopamine, propranolol, captopril, atenolol
      - 1. **Other:**
   3. **Stroke:** simvastatin, atorvastatin, aspirin, mannitol
      - 1. **Other:**
   4. **Diabetes:** glibenclamide, glucagon, insulin, metformin
      - 1. **Other:**
   5. **Respiratory medicines:** beclomethasone, budesonide, adrenaline, epinephrine, ipratropium bromide, salbutamol, prednisolone, hydrocortisone
      - 1. **Other:**
