# Supplementary material for: Analysis of rural health centres preparedness for the management of diabetic patients in Malawi
Source: BMC Res Notes. 2018 May 2;11:267. doi: 10.1186/s13104-018-3369-7 (PMC5932777; doi:10.1186/s13104-018-3369-7)
Supplement: Supplementary file 3 [file 13104_2018_3369_MOESM2_ESM.docx]

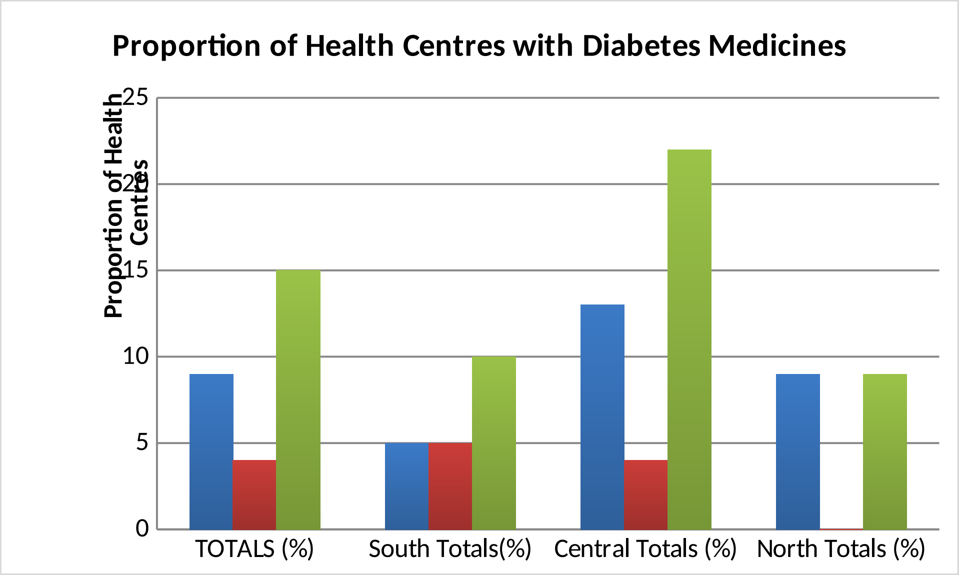

Proportion of Health Centres with Diabetes Medicines
Proportion of Health Centres
25
20
15
10
5
0
TOTALS (%)
South Totals(%)
Central Totals (%)
North Totals (%)
